# Gene Environment Interactions in the Etiology of Neural Tube Defects

**DOI:** 10.3389/fgene.2021.659612

**Published:** 2021-05-10

**Authors:** Richard H. Finnell, Carlo Donato Caiaffa, Sung-Eun Kim, Yunping Lei, John Steele, Xuanye Cao, Gabriel Tukeman, Ying Linda Lin, Robert M. Cabrera, Bogdan J. Wlodarczyk

**Affiliations:** ^1^Department of Molecular and Human Genetics and Medicine, Center for Precision Environmental Health, Baylor College of Medicine, Houston, TX, United States; ^2^Department of Molecular and Cellular Biology, Center for Precision Environmental Health, Baylor College of Medicine, Houston, TX, United States; ^3^Department of Pediatrics, The University of Texas at Austin Dell Medical School, Austin, TX, United States

**Keywords:** teratogen, gene X environment interaction, birth defect, neural tube defect, anti-epileptic drugs, arsenic

## Abstract

Human structural congenital malformations are the leading cause of infant mortality in the United States. Estimates from the United States Center for Disease Control and Prevention (CDC) determine that close to 3% of all United States newborns present with birth defects; the worldwide estimate approaches 6% of infants presenting with congenital anomalies. The scientific community has recognized for decades that the majority of birth defects have undetermined etiologies, although we propose that environmental agents interacting with inherited susceptibility genes are the major contributing factors. Neural tube defects (NTDs) are among the most prevalent human birth defects and as such, these malformations will be the primary focus of this review. NTDs result from failures in embryonic central nervous system development and are classified by their anatomical locations. Defects in the posterior portion of the neural tube are referred to as meningomyeloceles (spina bifida), while the more anterior defects are differentiated as anencephaly, encephalocele, or iniencephaly. Craniorachischisis involves a failure of the neural folds to elevate and thus disrupt the entire length of the neural tube. Worldwide NTDs have a prevalence of approximately 18.6 per 10,000 live births. It is widely believed that genetic factors are responsible for some 70% of NTDs, while the intrauterine environment tips the balance toward neurulation failure in at risk individuals. Despite aggressive educational campaigns to inform the public about folic acid supplementation and the benefits of providing mandatory folic acid food fortification in the United States, NTDs still affect up to 2,300 United States births annually and some 166,000 spina bifida patients currently live in the United States, more than half of whom are now adults. Within the context of this review, we will consider the role of maternal nutritional status (deficiency states involving B vitamins and one carbon analytes) and the potential modifiers of NTD risk beyond folic acid. There are several well-established human teratogens that contribute to the population burden of NTDs, including: industrial waste and pollutants [e.g., arsenic, pesticides, and polycyclic aromatic hydrocarbons (PAHs)], pharmaceuticals (e.g., anti-epileptic medications), and maternal hyperthermia during the first trimester. Animal models for these teratogens are described with attention focused on valproic acid (VPA; Depakote). Genetic interrogation of model systems involving VPA will be used as a model approach to discerning susceptibility factors that define the gene-environment interactions contributing to the etiology of NTDs.

## Introduction

Human congenital malformations resulting in physical or mental deficits are a leading cause of infant mortality. Estimates from the United States Center for Disease Control and Prevention (CDC) determine that close to 3% of all United States newborns present with birth defects; the worldwide estimate approaches 6% of infants presenting with congenital anomalies. The complications arise from birth defects can be devastating, with family expenses estimated at more than half a million US dollars. The scientific community has recognized for decades that the vast majority of birth defects have undetermined etiologies, although we propose that environmental exposures interacting with inherited susceptibility genes are major contributing determinants. Failure to properly form and fuse the developing neural tube during early embryogenesis results in significant morbidity and mortality and as such, these malformations will be the primary focus of this review.

## Neural Tube Development

The developing nervous system in vertebrates arises initially from the neural plate, an embryonic layer of cells that is specified and differentiates from the ectoderm immediately after gastrulation proceeds. The layer of cells located at the central portion of the neural plate will give rise to the central nervous system, while on its periphery, adjacent to the neural groove, neural crest cells will be transiently formed before delamination and migration to the adjacent paraxial mesoderm (Smith and Schoenwolf, 1997). The next step is neurulation, a morphogenetic process in which the neural plate will bend over itself forming the neural tube. The most anterior portion of this developing tube will form the cephalic structures of the central nervous system, while the spinal cord will be formed by the truncal and caudal portions located posteriorly on the tube. In a highly regulated manner following neurulation, the neural crest will migrate ventrally to colonize specific sites prior to differentiating into a range of diverse cell types (Smith and Schoenwolf, 1997).

In vertebrate embryos, the adult brain originates from the most rostral anterior portion of the developing neural tube, while the spinal cord originates from the most posterior caudal extremity. Failure during neural tube closure can occur in different regions during the developmental axis formation, resulting in different abnormalities. Anencephaly cases occur when the anterior section of the developing neural tube fails to properly fuse, resulting in incomplete development of both the skull and the brain. Failure of neural tube fusion in the most caudal portions of the embryo results in the malformation commonly referred to as spina bifida. This malformation is more common, and distinctions are made between closed (occulta) and open (aperta) defects. Spina bifida aperta involves exteriorized neural tissue that is not fully covered by skin. When a cystic protrusion is detected, there the two primary subtypes referred to as meningocele, when the protrusion involves the meninges along with cerebrospinal fluid, and myelomeningocele, when the cystic protrusion contains the portions of the developing spinal cord. Finally, closed spina bifida occulta is when the defect is covered by skin. Cases, where spina bifida occulta occurs, can evolve asymptomatically throughout life and might never be detected (Rowland et al., 2006; Copp et al., 2015; Avagliano et al., 2019).

When the embryonic neural tube closure reaches its final stage, it is possible to identify the neuroepithelium, which is composed by a monolayer of bipolar cells, delimiting the ventricular lumen. The basal region of the neuroepithelium contacts the basal lamina at the periphery of the tube, while the apical region is in contact with the lumen or ventricle. Neurons are generated only by the lateral regions of the neural tube, with the roof and floor plates responsible for the production of morphogens that standardize the different neural types along the dorsoventral axis. Within the primordial spinal cord, the neural tube can be functionally divided into a dorsal domain, with neurons that receive sensory information, and a ventral domain, with motor neurons (McConnell, 1995).

## Clinical Findings and Epidemiology

Neural tube defects (NTDs) are among the most prevalent of all human congenital anomalies. Fetuses with anencephaly and craniorachischisis typically do not survive to term, although a small proportion of anencephalic infants are viable for a limited time post-parturition (Greene and Copp, 2014). Unlike infants with anterior NTDs, those with meningomyeloceles are viable, yet are likely to suffer from significant disabilities. Worldwide NTDs have a prevalence of approximately 18.6 per 10,000 live births (Blencowe et al., 2018), while the prevalence of NTDs is 6 per 10,000 births in most regions of the United States (Shaw et al., 1994; Wallingford et al., 2013). There are in excessive of 3,000 NTD-compromised pregnancies annually in the United States, resulting in lifetime medical expenses estimated to exceed $560,000 per infant. Most investigators believe that NTDs have a multifactorial inheritance pattern that involves contributions from environmental (White et al., 1988; Brender and Suarez, 1990; Sever, 1995; Shaw et al., 1996, 1998, 1999a,b; Wasserman et al., 1998; Carmichael and Shaw, 2000; Brender et al., 2002; Farley et al., 2002; Carmichael et al., 2003; Lundberg et al., 2003; Suarez et al., 2003; Waller et al., 2007; Cabrera et al., 2019) and genetic elements (Campbell et al., 1986; Shaw et al., 1994; Blom et al., 2006; Steele et al., 2019). Data collected over the last 40 years demonstrates that the periconceptional use of folic acid reduces the population burden of NTDs (Group MMWR, 1991; Czeizel and Dudas, 1992; Berry et al., 1999; Honein et al., 2001; Williams et al., 2002; Canfield et al., 2005; De Wals et al., 2007), although the underlying developmental processes that benefit from the folic acid and reduces the NTD risks are not well understood. Clearly, the fortification of food supply did not make the problem of NTDs go away. There remain a large number of NTD cases that are born despite folic acid, with these folic acid-resistant NTDs occurring at an apparent baseline rate of 5 per 10,000 live births (Heseker et al., 2009). Thus, NTDs remain a substantial public health problem even in countries with mandatory folic acid food fortification.

### Nutritional Risk Factors for NTDs

Folate status is clearly established as a modifier of NTD risk, as mothers deficient in water-soluble vitamin B9 are more likely to have infants with NTDs (Blom et al., 2006; Wallingford et al., 2013). Folates derived from food are generally in a polyglutamate form, while folic acid is a highly stable synthetic form that is a monoglutamate. The monoglutamate form is initially metabolized in the liver, rather than in the intestines, which is the case for the naturally occurring folates. Since folic acid is physiologically inert in the human body and must be transformed by the enzyme dihydrofolate reductase (DHFR) to bioactive molecules including 5-methyltetrahydrofolate (5MTHF), this process is compromised by the relatively low levels of DHFR in the liver (Ferrazzi et al., 2020). This results in significant amounts of unmetabolized folic acid entering the systemic circulation. Folates are involved in multiple metabolic functions including transmethylation reactions, the regulation of homocysteine concentrations, and nucleic acid biosynthesis (Ferrazzi et al., 2020). Several clinically important pharmaceutical compounds with known teratogenic effects, including trimethoprim, Depakote (valproic acid, VPA), and methotrexate, interfere with folate metabolism by inhibiting DHFR, which limits the production of 5MTHF (Ferrazzi et al., 2020).

Folic acid deficiency is not the sole nutrient that, when deficient, has been associated with increased risk for NTDs. Maternal vitamin B12 deficiency is also a known risk factor for NTDs, as vitamin B12 is a co-factor of the enzyme methionine synthase, an important component of one-carbon metabolism (OCM) responsible for converting homocysteine to methionine. In an excellent review by Molloy (2018), the results of 24 different studies on the relationship of low maternal serum vitamin B12 levels and risk for NTDs make it clear that low maternal vitamin B12 status increases the NTD risk to the developing embryo. Furthermore, the risk appears to be independent of maternal folate status (Molloy, 2018). While the data is not as robust as it is for folic acid, in the five studies that come from larger cohorts (>80 NTD cases), which are global in nature and involve Canadian, United States, Chinese, and Tunisian populations, low maternal serum vitamin B12 status is linked to increased NTD risk. That said the literature is primarily populated by small studies with limited cohorts of NTD patients. For example, Senousy et al. (2018) reported on a cohort of 50 Egyptian mothers of infants with a NTD. Maternal serum levels of vitamin B12 were significantly decreased in NTD cases compared to controls, while homocysteine (Hcy) and methylmalonic acid (MMA) concentrations were elevated, demonstrating that low vitamin B12 status is a risk factor for NTDs. Ayaz and Asoglu (2020) reported from the Van province of northern Turkey that 33% of their mothers having infants with NTDs were vitamin B12 deficient. In a study, Fofou-Caillierez et al. (2019) described a cohort with a significant decrease (33.3%) in vitamin B12 concentration along with a 58.6% reduction in SAM among tissues harvested from NTD compared with non-NTD liver tissues. These investigators described a 2.2-fold reduction in vitamin B12 levels in cord blood from NTD fetuses compared to unaffected controls. The decreased vitamin B12 concentration may represent reduced bioavailability in the NTD fetuses or reduced maternal levels (Kirke et al., 1993; Li et al., 2016). It has also been reported that women consuming diets rich in vitamin B6 and B12, choline, and methionine had lower NTD risks among their progenies. Clearly, a diet rich in one-carbon analytes reduces the risk for birth defects such as NTDs (Petersen et al., 2019).

### Genetic Risk Factors for NTDs

NTDs are believed to result from multiple factors with both genetic and environmental contributions (Copp et al., 2013; Wallingford et al., 2013; Ross et al., 2017). Human epidemiological evidence for a genetic component derives from the strong concordance of NTDs between monozygotic twins (7.7%) compared to like sex/dizygotic twins (4.0%; Shaw et al., 1994; Wallingford et al., 2013; Blencowe et al., 2018). Furthermore, while neural tube closure defects can be familial after one NTD affected pregnancy, the recurrence risk is 1 in 20, and even after two affected pregnancies, the recurrence risk does not exceed 10%, strongly arguing against a monogenic causation. A number of case-control studies focusing on one, or at most, a few candidate genes have been used to identify alleles suggestive of an association with increased NTD risk. On the strength of folic acid NTD prevention studies, the interrogation of a thermolabile variant (C677T) in the 5-MTHFR gene was proposed to increase the NTD risk (Shields et al., 1999) in some, but not all, studied cohorts. Candidate genes selected either on the basis of mouse studies or trends in human genome sequences of relatively small case numbers have been replicated in human targeted resequencing studies for a number of genes, including many in the planar cell polarity (Kibar et al., 2007; Robinson et al., 2012; Lei et al., 2013, 2014, 2019) or WNT signaling pathways (Lei et al., 2015).

The well documented over 240 genes, whose mutation cause NTDs in the mouse (Harris and Juriloff, 2010), support the likelihood that numerous gene defects contribute to NTDs. Mouse genetic studies have also provided the insight that genetic background significantly effects the penetrance of NTDs in individual mice harboring those previously identified mutations and modifier loci that have been mapped in several mutant lines (Juriloff et al., 2001; Korstanje et al., 2008). Most null murine mutants (>90%) present as fetuses with many affected developing organs with high penetrance in homozygotes, while some mutations cause NTDs in digenic, trigenic, and oligogenic combinations, an etiology that is consistent with the genetic causation described in human NTD patients (Chen et al., 2018; Wang et al., 2018), as human NTDs most often arise through an omnigenic interplay of deleterious genetic variants and environmental factors influencing the function of core pathways such as OCM (Boyle et al., 2017; Chen et al., 2018). What is important to note is that, in spite of strong data documenting, the role of genetic factors in the etiology of NTDs, there are no clinically actionable NTD candidate genes known at this time that influence the management of high risk pregnancies. The advent of next generation sequencing (NGS) opens up greater possibilities of dissecting out the genomic architecture underlying NTDs in the coming years.

### Teratogens Associated With Inducing Neural Tube Defects

#### Polycyclic Aromatic Hydrocarbons

Polycyclic Aromatic Hydrocarbons (PAHs) are commonly found environmental pollutants that are believed to be risk factors for NTDs. PAHs enter the environment following the incomplete burning of biomass and are generally recognized for their grave potential to adversely impact human health. Multiple PAH compounds are considered to be either carcinogenic, mutagenic, and/or teratogenic (Pashin and Bakhitova, 1979). Animal experiments have consistently demonstrated that benzo(a)pyrene-7,8-dihydrodiol-9,10-epoxide, a i metabolic derivative of benzo(a)pyrene, is capable of producing multiple types of congenital malformations in exposed mouse embryos including: NTDs, gastroschisis, and phocomelia (Barbieri et al., 1986). With respect to human PAH teratogenicity, there have been many epidemiological studies reporting that maternal exposure to PAHs is responsible for an elevated risk of NTDs. In an interesting study conducted in the United States, it was noted that women who are either height and weight proportionate or underweight and are gestationally exposed to PAHs had more NTD affected infants than expected (Langlois et al., 2012). Another study conducted in Shanxi Province of northern China reported that indoor cooking and heating during the periconceptional period put mothers at an elevated risk for having NTD affected offspring (Liu et al., 2016).

Wang et al. (2015) described a potential association between the concentration of PAHs c in maternal serum and an increased risk for birth defects including NTDs. Their study was based on a case-control design and how the energy usage by households as well as lifestyle parameters impacted PAH exposure. The study involved mothers from Shanxi Province in China who had NTD-complicated pregnancies (*n* = 117) and 121 control mothers of infants without any malformations. At the time of delivery or pregnancy termination, a blood sample was drawn, and multiple PAHs were analyzed by gas chromatography-mass spectrometry. They determined that the levels of 13 different PAHs differed significantly in the cases than in the controls. A well-defined dose-response relationship was evident between the concentrations of PAHs and the increased risk for an adverse pregnancy outcome such as an NTD. With respect to NTD risk, it was determined that the high-molecular-weight PAHs (H-PAHs) had a greater impact than low-molecular-weight PAHs (L-PAHs). Thus, maternal exposure to PAHs is considered to be a risk factor for NTDs, and that select H-PAHs are associated with a greater NTD risk than are L-PAHs (Wang et al., 2015).

A possible association between the aryl hydrocarbon receptor (AHR) and select metabolic enzyme variants as determinants of NTD risk has been under investigation (Wang et al., 2014). Cytochrome P450 (CYP) enzymes CYP1A1, CYP1A2, and CYP1B1, which are members of the phase I metabolic enzyme family, are involved in the metabolic activation of PAHs to epoxide intermediates, prior to their conversion into diol-epoxides. There have been a number of single nucleotide polymorphisms (SNPs) in human genes coding for these enzymes that result in significant modifications of their normal enzymatic activities. After collecting blood samples from 534 mothers who conceived newborns or fetuses presenting with NTDs as well as from 534 control mothers who had healthy newborns, they interrogated the samples for 12 polymorphisms in the AHR and cytochrome P450 (CYP) genes. They determined that the CYP1B1 rs2855658 GG variant can modify the effect of indoor air pollution on NTD risk (Wang et al., 2014).

The AHR is a transcription factor that is a member of the BHLH superfamily, with a relatively wide and open Ligand Binding Domain (LBD), which can be activated in response to environmental stimuli such as pollutants, xenobiotics, and oxygen levels. Once activated AHR mediates induction of the detoxifying enzymes CYP1A1 and CYP1B1 (Noda et al., 2003). Intriguingly, Zalc et al. (2015) established the importance of Pax3 and Pax7, two essential transcription factors required for normal cranial neural crest cell development, on the regulation of the environmental stress response pathway mediated by AHR signaling (Zalc et al., 2015). Pax 3 variants are well-established risk factors for NTDs (Wallingford et al., 2013). Impacting the expression of critical transcription factors that compromise AHR signaling will no doubt inhibit cellular responses that can compromise normal embryonic development. These results are consistent with the demonstration that aberrant hyper-methylation of the Pax3 gene, which leads to its downregulation after PAH exposure, is associated with increased NTD risk in humans (Lin et al., 2019a).

#### Arsenic-Induced Neural Tube Defects

Inorganic arsenic (Asi) is a natural environmental contaminant in drinking water, air, and food in the form of arsenate [pentavalent, As (V)] or arsenite [trivalent, As (III)]. Arsenic has many agricultural applications as a pesticide or herbicide, and it is even used therapeutically for the treatment of multiple human diseases. Arsenic is more widely recognized for this toxicity. Asi is believed to be a risk factor for different types of cancer (Smith et al., 1992). There is also some evidence that Asi is a human teratogen. Despite the data presented in a number of epidemiological investigations, a universal acceptance of Asi as a source of human congenital malformations is not yet established (Holson et al., 2000; DeSesso, 2001). As one might imagine, performing such epidemiological studies can be challenging and as a result, the limited number of women with *in utero* Asi exposure was generally too small to reveal significant associations with specific malformations. Furthermore, most of these human epidemiological investigations required the use of proxy measures of exposure, which could potentially reduce the reliability of the data and lead to subject misclassification (Holson et al., 2000).

Acute high dose *in utero* arsenic exposure is a risk factor for pre- and post-natal mortality (Lugo et al., 1969; Bolliger et al., 1992). Furthermore, chronic maternal low dose arsenic exposure has been tied to increased pre- and post-natal mortality, as well as low birth weight and developmental disabilities (Shalat et al., 1996). The small cohort size compromises the ability to statistically demonstrate positive associations with individual birth defects, although multiple investigations revealed an association between maternal arsenic exposure to increased prevalence of congenital malformations in their offspring. A case-control study in Texas focused on maternal heavy metal exposures and birth outcomes revealed trending odds ratios for an increased risk of NTDs specifically with arsenic exposure (Brender et al., 2006). Although not statistically significant for reasons previously mentioned, a potential association between maternal arsenic exposure and NTD risk cannot be excluded. There is also indirect evidence that demonstrating As as an NTD risk factor stemming from epidemiological studies of pesticides. Several such studies indicate that NTD risks increase with maternal exposure to pesticides or if pregnant women reside close to agricultural areas (Shalat et al., 1996; Lacasana et al., 2006; Rull et al., 2006). The heightened concern over environmental arsenic as a birth defect risk factor will continue given that arsenic remains to be used both industrially and in agricultural practices.

Much of the ambiguity over human birth defect risks from environmental arsenic exposure has changed over the last two decades. Research conducted globally has provided strong evidence linking maternal arsenic exposure with an increased risk for NTD affected offspring. Studies conducted in Bangladesh have been particularly informative, given that Bangladesh has one of the highest prevalence rates of NTDs in the world (Mazumdar, 2017), and the large segments of the Bangladeshi population suffers from excessive exposure to arsenic from drinking well water (Mazumdar et al., 2015; Kancherla et al., 2017; Obrycki et al., 2019). As arsenic is methylated during its biotransformation, there is a concern that in populations that are highly exposed to arsenic, folate status would be an important confounding variable with respect to NTD prevalence. As the linkage between folic acid and NTDs is well-established, it made sense to look for NTDs in population studies from the same region. They determined that mothers receiving supplemental folic acid significantly lowered the risk for NTDs in the offspring (OR = 0.42, CI 0.18–0.96), thus validating the protective association between folic acid status and NTDs in Bangladeshi infants (Kancherla et al., 2017).

There is a wealth of experimental studies to evaluate the teratogenicity of arsenic in various experimental animal species. These studies utilized multiple arsenic forms, the compounds were administered *via* differing routes and investigators took advantage of different study designs. What was apparent from these investigations was the very consistency of positive findings of teratogenicity when experimental animals are exposed *in utero* to arsenic. Most notable among the malformations observed were neural tube and craniofacial defects (Wlodarczyk et al., 1996; Golub et al., 1998; Holson et al., 2000; Hill et al., 2008). Hill et al. (2008) reported that oral administration of arsenic to pregnant days significantly increased the rate of exencephaly in mice in the absence of any observed maternal toxicity. There was a distinct dose response for the arsenic induced NTDs, with abnormal fetuses being detected even at the lowest treatment doses of arsenic used in these studies (Hill et al., 2008). While these dosages might appear to be extremely high when compared with human environmental exposures such as those in Bangladesh, it is critically important to understand that when conducting laboratory animal developmental toxicity studies, it is prudent to consider working with these higher dosages while being focused on the presence of any maternal toxicity when drawing any meaningful conclusions.

Given the widespread distribution of environmental arsenic, the susceptibility of humans to arsenic toxicity and the fact that orally administered arsenic was teratogenic in the study of Hill et al. (2008) suggests a potential link between human arsenic exposure and elevated risks for NTDs. This provides sound reason to perform future sophisticated human epidemiological studies to determine if environmental arsenic exposure poses a significant teratogenic threat to exposed human populations as well as genetic interrogations to help to define genetically susceptible population.

#### Pesticide-Induced Neural Tube Defects

Human exposure to pesticides can occur as a result of either occupational exposure, environmentally, or through food/water consumption of pesticide residues post-application of these compounds (Kalliora et al., 2018). As pesticides are literally designed to be lethal to a range of agricultural pests, they are believed to work through various combinations of toxicological mechanisms, including being endocrine disruptors, immunotoxicants, or neurotoxicants, which tends to target the mammalian nervous system, which is especially sensitive to these agents. Organophosphate pesticides have been shown to be particularly harmful to humans when exposure occurs prenatally or during early childhood (Munoz-Quezada et al., 2013). While it is challenging to link NTDs to specific pesticides, many of the organophosphates, chlorpyrifos, and vinclozolin are believed to have such a teratogenic potential (Stillerman et al., 2008). As with other suspected teratogens, the human epidemiological record is seldom consistent or remarkably in depth. Differences in study design and exposure assessment accuracy, which relies on self-reporting, are thought to underlie the different study outcomes, along with limited cohort sizes and complex exposure mixtures (Kalliora et al., 2018). Maternal exposure to endosulfan, DDT, and dichlorodiphenyldichloroethylene (DDE) has been positively associated with an increased risk for NTDs in exposed offspring (Kalra et al., 2016). Women who have been exposed to these compounds are at an 11-fold increased risk of having an NTD affected pregnancy compared to unexposed control mothers (Kalra et al., 2016). Clearly, there is much more work to be done with respect to the suspected or confirmed teratogenicity of many agriculturally significant pesticides.

#### Maternal Hyperthermia-Induced Neural Tube Defects

For the past 30 years, there have been several investigations into the role maternal hyperthermia may contribute to the population burden of NTDs. Maternal hyperthermia can be the result of febrile disease, exposure to heat sources occupationally and from the environment, which includes electromagnetic radiation and ultrasound sources (Edwards, 2006). While there is no clear consensus as to the overall impact of maternal hyperthermia on the risk of having an NTD affected pregnancy, the majority of epidemiological studies have reported a positive association (Layde et al., 1980; Kurppa et al., 1991; Milunsky et al., 1992; Zhang and Cai, 1993; Lynberg et al., 1994; Shaw et al., 1998; Botto et al., 2002; Suarez et al., 2004). The lack of consistency in the outcome of these studies are likely due to multiple factors, including differing study designs, limited size of the NTD cohorts and heterogeneity of the presenting maternal illness. It is important to also recognize that in Western Societies, when women have a febrile disease, they are often taking antipyretics and other medications that complicate dissecting out the role of the maternal fever from that of the medications on increasing the risk for NTDs. In one of the larger investigations that relied upon a population-based case control study that included 653 NTDs, Shaw et al. (1998) determined that elevated risks for NTDs were found when mothers had febrile illnesses during the first trimester. They also reported that when febrile mothers took acetaminophen, the NTD risk was actually lowered (Shaw et al., 1998). It was also interesting to note that there was a greater risk for anencephaly than for posterior defects (Milunsky et al., 1992). Li et al. (2007) in Beijing using a similar study design as that used in California (Shaw et al., 1998) reported that NTD risks were significantly associated with maternal febrile illness or flu (AOR = 3.93, 95% CI: 2.48–6.23) consistent with the Shaw study. However, these investigators reported that women taking antipyretics for their febrile disease had a higher adjusted odds ratio for anencephaly than for spina bifida. Clearly, maternal fever together with medications used to reduce the fever resulted in an increased risk for NTD affected pregnancies (Li et al., 2007).

Kerr et al. (2017) ascertained a total of 375 NTD cases and 8,247 non-malformed controls for their study cohort. Case mothers were more often overweight or obese to have illnesses during pregnancy that did not induce febrile episodes and to meet the recommended daily folic acid intake when compared to control mothers. Case women who had infants with NTDs reported having febrile disease during pregnancy more commonly (5.1%) than did the mothers of control infants (1.9%; Kerr et al., 2017). Among the 2.4-fold increased NTD risk among women suffering from febrile disease while pregnant, it was found that folate replete women had a significantly lower risk for an NTD affected infant than did women with fevers who did not have sufficient folate levels during pregnancy (OR = 3.4; 95% CI: 0.8–4; Kerr et al., 2017). Analyzing data obtained from the National Birth Defects Prevention Study, which was multi-year case-control epidemiological survey of congenital malformations in the United States involving telephone interviews of case mothers (*n* = 17,162) and controls (*n* = 10,127), Waller et al. (2018) determined that there was a significant association with three different NTDs (anencephaly, spina bifida, and encephalocele) and four additional types of birth defects among mothers who reported a fever during early pregnancy. From this study, it was apparent that it was the fever itself, and not the underlying disease, that elevated the risk for NTDs (Waller et al., 2018).

The mechanism by which maternal fevers during early gestation disrupt normal NTC has not been adequately resolved to date. There is, however, a significant experimental animal literature related to the impact of maternal hyperthermia on the processes involved in NTC. It is a generally well accepted axiom that maternal core temperature increases above 2°C for long periods of time or increases greater than 4°C for shorter intervals can produce structural malformations in experimental animals (Ziskin and Morrissey, 2011). The initial experimental studies were conducted in guinea pigs by an Australian group lead by Prof. Marshall Edwards, who reported miscarriages and newborns with arthrogryposis (Edwards et al., 2003). The adverse endpoints in experimental model systems were not restricted to arthrogryposis, as hyperthermic exposure can cause the usual spectrum of teratogenic endpoints, including embryolethality, developmental delay/growth retardation, and structural malformations. It has also been observed that maternal hyperthermia exposure is capable of disrupting normal development if the heat exposure is high enough, and it occurs during a sensitive period of development. For example, during early embryogenesis, hyperthermia can alter cellular kinetics and leads to a lack of proliferating cells, delays differentiation of cells, enhances apoptosis, and compromises the developing embryonic vascular system (Waller et al., 2018). With respect to NTDs, a brief exposure in mice, rats and guinea pigs beginning prior to and during NTC can produce such malformations (Edwards, 1967). When exposure occurs after the most susceptible period, while it may not induce NTDs, it can still result in adverse pregnancy outcomes. In animal experiments, the congenital defect that is produced depends on the species that is being exposed to heat and precisely when the exposure occurs during morphogenesis. Furthermore, by utilizing inbred mouse strains, it is possible to identify highly susceptible and highly resistant strains, reflective of the genetic background of the given strains (Finnell et al., 1986). In one notable experimental system, the pregnant dam of different inbred strains was placed in a 50-ml conical tube with holes drilled in the side of the tube to allow water to circulate and then suspended in a 43°C waterbath for 10 min (Finnell et al., 1986). Depending on the strain/genotype of any given embryo, the response frequency of NTDs would vary widely. For example, the DBA/2J mice were completely resistant to the induction of exencephaly by this heat treatment, while SWV embryos exposed to an identical treatment presented with near 100% penetrant NTDs (Finnell et al., 1986). By performing linkage studies in an attempt to identify the responsible sensitivity genes, Finnell et al. determined that the sensitivity to hyperthermia induced NTDs was lost in the F1 generation after the first crosses between the resistant C57BL/6 J strain with the SWV strain. Backcrosses to the maternal SWV strain partially restored some of this sensitivity to hyperthermia induced NTDs (Finnell et al., 1986). Thus, the complex nature of gene-environment interactions requires the development of more sophisticated approaches utilizing the tools of contemporary molecular biology to dissect out significant contributing susceptibility factors to the etiology of environmentally induced NTDs.

## Discussion

### Identification of Susceptibility Genes Associated With Anti-seizure Medications Induced NTDs Using Mouse Models

Anti-seizure medications (ASMs) have long been a source of concern when used by pregnant women. The initial association between ASMs and an increased risk for congenital malformations dates back to a short publication in German in 1963 following the recognition of Thalidomide’s teratogenic potential (Mueller-Kuppers, 1963). A robust literature that has developed over the last 50 years succinctly documents the apparent teratogenicity of all the frontline ASMs commonly used to manage seizure disorders (Dansky and Finnell, 1991). These ASMs include phenytoin (Hanson and Smith, 1975; Hanson et al., 1976; Buehler et al., 1990) trimethadione (Zackai et al., 1975), carbamazepine (Ornoy and Cohen, 1996; Hill et al., 2010; Kohl et al., 2019), lamotrigine (Hernandez-Diaz et al., 2012; Tomson et al., 2019; Trifu et al., 2020), levetiracetam (Tomson et al., 2015; Kuo et al., 2020), VPA (Wyszynski et al., 2005; Koren et al., 2006; Kluger and Meador, 2008; Gerard and Meador, 2015), and topiramate (Veroniki et al., 2017; Vajda et al., 2020). More alarming is the fact that many of these compounds are now widely prescribed to women of reproductive age for the management of more common afflictions, including neuropathic pain, migraine headaches, mood disorders, obesity, and psychiatric disorders, adding significantly to the number of women of reproductive age who are exposed to these important medications. There are believed to be 1.5 million United States epileptic women of childbearing age responsible for 3–5 infants per 1,000 live born (Harden et al., 2009; Hill et al., 2010). This is a gross underestimate of the number of women of reproductive age exposed to ASMs annually, given their therapeutic application to treat more prevalent disorders as described above. Recent studies have confirmed that there were in excess of four million prescriptions of anti-seizure medications provided annually to United States women between 15 and 40 years of age (Adedinsewo et al., 2013). While the prevailing consensus in the literature is that changes in prescribing trends globally has shifted to safer, less teratogenic ASMs, the clinical concern is accordingly lessened (Hurault-Delarue et al., 2019). In fact, ASM usage and exposure during pregnancy continues to rise from 15.7 to 21.9 per 1,000 deliveries over a 10-year period ending in 2009 (Bobo et al., 2012). After nearly 60 years of study, there does not appear to be any ASM that is completely free of a teratogenic potential. The literature is replete with studies documenting the teratogenicity of all the commercially available ASMs (Viinikainen et al., 2006; Palac and Meador, 2011). Major congenital malformations (MCMs) secondary to *in utero* ASM exposure is a consistent finding in some but not all exposed infants (Meador et al., 2006; Palac and Meador, 2011). The most commonly reported birth defects following maternal use of AEDs include craniofacial, cardiac, skeletal, urologic, and NTDs (Meador et al., 2006; Palac and Meador, 2011). A comprehensive review of the teratogenicity of all ASMs is beyond the scope of this review. Attention will be focused on just two compounds-VAP (Depakote) and Topiramate (TPM; Topamax). Depakote is widely utilized worldwide and is associated with the most significant teratogenic threat to developing infants of all the commercially available ASMs (Perucca, 2002; Meador et al., 2006; Viinikainen et al., 2006). The prospective Neurodevelopmental Effects of Antiepileptic Drugs Study reported that serious adverse pregnancy outcomes-either major structural birth defects or miscarriage occurred in 20% of VPA exposed infants, again validating the consensus that Depakote represents the most teratogenic of the existing frontline ASMs (Meador et al., 2006). The International Registry of Antiepileptic Drugs and Pregnancy reported that 18% of the offspring of mothers receiving Depakote in their extensive cohort presented with MCMs in a dose-response manner (Tomson et al., 2011). It has been estimated that the total costs associated with children exposed *in utero* to ASMs requiring medical care or educational interventions now exceed $1.8 billion per year. With respect to NTDs and the treatment of women of reproductive age with Depakote, the most important clinical question concerns the benefit of concurrent high does folic acid therapy. To date, with the possible exception of neurocognitive outcomes (Meador et al., 2020), there is no evidence demonstrating a protective benefit from high dose folic acid supplementation to reduce the risks of an NTD (Hill et al., 2010).

Another clinically important ASM for which there is significant teratogenic concern is Topamax® [Topiramate; “TPM”; 2,3:4,5-di-O-isopropylidene-(3-D-fructopyranose sulfamate)], a second-generation anti-epileptic medication predominantly not only used in the treatment of seizure disorders but also having therapeutic applications, on and off label, in the treatment of migraine headaches, neurobehavioral disorders, and obesity. Post-marketing surveillance conducted by the pharmaceutical company and advanced information provided by anticonvulsant drug pregnancy registries provided indications that congenital defects, including hypospadias, cleft palate, NTDs, and microcephaly, were being observed in infants exposed *in utero* to TPM monotherapy. Pregnancy data from the United Kingdom Epilepsy and Pregnancy Registry also revealed infants with cleft lip and palate and hypospadias among 28 informative pregnancies with TPM monotherapy exposure (Morrow et al., 2006). The major congenital malformation rate consequently was 7.1% and the odds ratio for MCM was 2.75 (95% Cl: 0.62–12.20). TPM’s odds ratio for major congenital malformations exceeded the ORs for Tegretol, Lamictal, Dilantin, Neurontin, and Keppra. Hunt et al. (2008), relying upon pregnancy outcome data from the United Kingdom’s Epilepsy and Pregnancy Register, described two cases of oral clefting in a small cohort of infants exposed to TPM monotherapy. The rate was 11-fold greater than that of the general population. There was also a single infant with hypospadias exposed to TPM monotherapy (14-fold increase over the general population), for an overall MCM rate of 4.8%, which is well within the range for most of the frontline anti-seizure medications, as women on ASMs have birth defect rates that exceed those of the general population (Kaneko et al., 1999; Samren et al., 1999; Kaaja et al., 2003; Artama et al., 2005; Morrow et al., 2006; Mawer et al., 2010; Blotiere et al., 2019; Vajda et al., 2020). Overall, the MCM rate for monotherapy exposure to TPM is similar to firstgeneration ASMs and consistently higher than other second-generation ASMs. A final note concerning the teratogenicity of TPM that deserves mention is the recurrence rate of congenital malformations in compromised pregnancies. Campbell et al. (2013) reported that the recurrence risk of birth defects was 33% for TPM monotherapy and 66% when TPM is used as part of a polytherapy treatment protocol. Although the cohort size of TPM exposed women was small, it is another signal that this medication should be avoided in women considering or who might possibly become pregnant while on the medication (Campbell et al., 2013).

**Figure 1:**
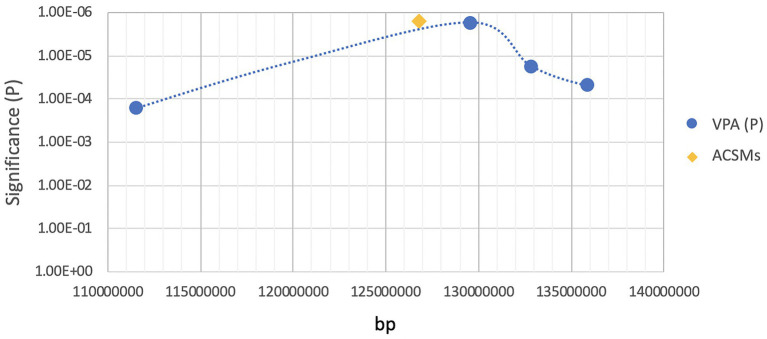
Location of ACSM family genes in the region of high sensitivity to valproic acid (VPA)-induced neural tube defects (NTDs).

As ASMs are widely prescribed medications, lacking a clear understanding of their teratogenic mechanisms action represents a challenging data gap that limits our ability to safely manage these high-risk pregnancies as well as our ability to design safer and efficacious ASMs. Our laboratory has been actively involved in experiments trying to better understand the gene-environment interactions that contribute to AED-induced birth defects. The focus of our teratogenic experimentation has been on VPA and its many analogs (Finnell et al., 1988, 1997; Wlodarczyk et al., 1996; Sobol et al., 2006; Shimshoni et al., 2007, 2008, 2010; Pessah et al., 2009, 2010, 2011; Kaufmann et al., 2010; Hen et al., 2013; Shekh-Ahmad et al., 2014a,b; Mawasi et al., 2015; Lin et al., 2019b). In trying to develop appropriate genetic model systems, we have primarily made the use of the inbred mouse strains, SWV/Fnn, which was highly sensitive to VPA-induced NTDs, and C57BL/6J, a resistant strain, where fewer than 10% of the exposed embryos had NTDs. We performed linkage analysis and fine gene mapping experiments in order to identify susceptibility loci responsible for VPA-induced NTDs. Using days from these two highly inbred mouse strains that received sodium valproate (600 mg/kg) *via* intraperitoneal injection on gestational day E8.5, the embryos failed to close their neural tubes in a strain-dependent manner (Lundberg et al., 2004). Further interrogations of the genomes from 131 backcrossed fetuses with NTDs revealed a major gene mapped to *D7Mit285* (*p* < 2 × 10^−6^), exceeding the threshold for significant linkage. We determined that recombination events had occurred in the chromosomal region located between *D7Mit285* and *D7Mit101*, which cover a 3.3-Mb region. This helped to establish the presence of a chromosomal region that contained a major gene or genes that regulates susceptibility to VPA-induced NTDs in mice (Figure 1; Taiwo et al., 2020). More robust genomic tools can now be applied to further refine this region of interest and better define those genetic factors regulating sensitivity to VPA’s teratogenicity in mice.

### Understanding the Mechanism of Action for VPA Teratogenicity

The current literature suggests that, while anticonvulsants may share their mechanisms in terms of anti-epileptic and toxic effects, they seem to differ in their mechanisms of teratogenicity, although the latter is largely unknown. Reports in the literature suggest that there are differing mechanisms of action underlying the efficacy for seizure control from that responsible for inducing birth defects (Löscher, 1999). It is likely that the teratogenicity, toxicity, and anticonvulsant effect of VPA are the direct effect of the drug and not its metabolites. Once administered, Depakote is biotransformed into multiple physiologically active compounds; however, given their limited concentration, they do not significantly contribute to the efficacy of the seizure control (Löscher, 1999). What is interesting about the teratogenicity of VPA is the relationship between its potency and the structural requirement that the molecule contain the following: an alpha-hydrogen atom, a carboxyl function and branching on C-2 with two chains containing three carbon atoms each for maximum activity (Nau, 1994). Several anticonvulsant mechanisms for VPA have been suggested to given its ability to be efficacious for many different epileptic diseases. VPA potentiates GABAergic functions, attenuates amino acidergic neuronal excitation induced by NMDA-type glutamate receptors and alters dopaminergic and serotonergic functions. As far as hepatic metabolism is concerned, VPA inhibits CYP (cytochrome P450) and UDPGT (uridine diphosphate glucuronosyltransferase) enzymes, while the other common anti-epileptic compounds – phenytoin, phenobarbital, primidone, and carbamazepine actually induce the production of these enzymes (Tanaka, 1999). Meanwhile, the most commonly used anticonvulsants are eliminated by hepatic metabolism and catalyzed by the enzymes CYP2C9, CYP2C19, CYP3A4, and UDGPT. The teratogenic mechanism for VPA is not well understood. Hypotheses for the teratogenicity of VPA include: interference with folate metabolism, embryonic lipid metabolism (Clarke and Brown, 1987), Zn metabolism (Wegner et al., 1990), neurotransmitter metabolism, altering the methylation of nucleic acids, post-translational methylation, the availability of methyl groups for other important cellular reactions, lowering embryonic pH value, the metabolism of VPA *via β*-oxidation leading to CoA sequestration, an increase in levels of reactive oxidative stress molecules, and the modulation of chromatin structure secondary to its negative impact on endogenous histone deacetylases (Hsieh et al., 2012). Clearly there have been many possible explanations reported for the teratogenicity of VPA.

VPA exposure is known to increase reactive oxidative species (ROS) production and leads to an increased frequency of homologous recombination (Sha and Winn, 2010). At this time, VPA inhibition of HDACs is believed by many investigators to be the principal way in which the teratogenicity of this anticonvulsant drug is mediated (Gurvich et al., 2005). This inhibition results from the binding to the catalytic center, which restricts substrate access, resulting in and hyper-acetylation of the N-terminal tails of histones H3 and H4 *in vitro* and *in vivo*. Inhibition of HDAC results in an overall increase in gene expression. Using *Xenopus* and zebrafish as model organisms, Gurvich et al. (2004) found that VPA exposure increased neural patterning and cardiac malformations. These defects were observed with transcriptional changes that were closely paralleled by those found in structurally unrelated HDAC inhibitors such as trichostatin A (TSA). VPA and its HDAC inhibiting analogs along with TSA had comparable effects on gene expression across a wide dose range in both model organisms studied, providing strong evidence that VPA exerts its teratogenic effects *via* HDAC inhibition (Gurvich et al., 2004).

Interaction of VPA with folate metabolism has long been suspected of underlying VPA’s teratogenicity and this hypothesis is among the best characterized to date. It has been established that plasma folate and methionine levels are significantly reduced upon VPA treatment, accompanied by an increase in homocysteine and tetrahydrofolate levels (Wegner and Nau, 1992). When VPA treatment is accompanied by folate supplementation, the exencephaly rates decreased by 50% in both mice and rats (Trotz et al., 1987). In humans, as described above, although it is known that folic acid intake can reduce NTDs by 50% (Werler et al., 1993; Shaw et al., 1994), there is no evidence that this is effective in preventing VPA-induced NTDs (Jentink et al., 2010; Ban et al., 2015). There have been several different hypotheses offered with respect to the impact VPA has on folate metabolism. However, one area that has received much less attention is the ability of VPA to directly inhibit the ability of folate receptors to bind and transport folic acid, therefore lowering serum folate concentrations, which may have significant teratogenic consequences.

Fathe et al. (2014) explored the binding affinities of three folate compounds (folic acid, s-folinic acid, and 5-methyltetrahydrofolate) to the folate receptors [folate receptor *α* (FRα; Folr1), folate receptor β (FRβ; Folr2), and the bovine folate binding protein (bFBP)]. These studies were conducted in both the presence and absence of VPA. The addition of VPA at IC50 concentrations significantly reduces receptor affinity for folates. The non-competitive nature of this interaction with VPA is clear, as increasing the concentration of VPA prevents the receptor from achieving signal saturation (Fathe et al., 2014). These investigators collected supernatant from HEK293T cells that were previously folate starved and then exposed to either folate or folate and VPA, to see how much of the folates would bind to cell surface folate receptors. As the VPA concentration of VPA was increased, there were significantly less folates bound to the cells (Fathe et al., 2014).

### Newer Methodologies, Newer Models, and Better Data

Despite decades of investigation, the etiology of NTDs remains to be clearly elucidated. One of the main reasons for this data gap is the lack of suitable models with which to study early developmental events directly in human embryos (Wallingford, 2005; Lei and Finnell, 2016). Fortunately, recent technological breakthroughs in the use of neural tube organoids provide a novel three-dimensional (3D) model system that enables us to better understand the development of the human neural tube in an *in vitro* system. Ideally of course, it is best to test hypotheses in human cells, although this is not always possible. As a result, we have relied upon the use of animal model systems as a reasonable surrogate, although this approach is far from perfect. By using animal models such as the mouse, chick, *Xenopus*, and zebrafish, investigators have successfully constructed multiple NTD models with which to probe at the cellular level the underlying mechanisms of failed neural tube closure (Hildebrand and Soriano, 1999; Gray et al., 2009; Manojlovic et al., 2014; Sedykh et al., 2018). However, it is important to be cognizant of the fact that the formation of the neural tube includes some differences among different model species, often with respect to the number of initiating closure sites and the timing and sequence of the closure proper. While NTC in mouse embryos closely approximates human NTC, there are still some differences between mouse and human. NTC in human embryos is initiated from only two closure sites, which is equivalent to the closure point 1 and 3 in mice of which there are four closure sites (Golden and Chernoff, 1993; O’Rahilly and Muller, 2002). In chick embryos, there are also two initial closure points, located at the future midbrain and at the hindbrain/cervical boundary. These closure sites undergo a bi-directional closure process (Golden and Chernoff, 1993; Nikolopoulou et al., 2017). Finally, in *Xenopus laevis*, NTC occurs almost simultaneously along the entire body axis (Runnels and Komiya, 2020).

Given these species-specific differences, the use of animal models has their limitations with respect to fully recapitulating the morphogenetic processes involved in human NTC. With the recent emergence of organoid culturing, it is now possible to create *in vitro* 3D cell models for NTC from human pluripotent stem cells (hPSCs). These neural tube organoids to a great extent simulate the *in vivo* cell composition of the neural tube and obviate the need to use model organisms for *in vivo* experimentation. This new approach makes it possible to study human neural tube development utilizing an *in vitro* culture system. It is also possible to create organoids from genetically modified mouse ESCs, which can serve as important proof of principle studies on specific gene variants. Neural tube organoid culture is based on the self-organizing ability of stem cells grown under extracellular matrix conditions in the presence of essential exogenous signaling factors. This is possible with both hiPSCs/hESCs and murine ESCs. Such cells have been created that maintain not only the proper dorsal-ventral organization (Meinhardt et al., 2014; Zheng et al., 2019), but also rostro-caudal pattern (Rifes et al., 2020) similar to that of a human neural tube that develops *in vivo*. Efforts have also been made to use variants of typical cell culturing equipment to essentially create organoids-on-a-chip. These advances offer greater stability of the neural tube phenotype as a result of enhanced precision and control of the *in vitro* microenvironment (Demers et al., 2016; Ranga et al., 2016; Park et al., 2019; Rifes et al., 2020). As it is possible to utilize single cells to create neural tube organoids, it opens up a new world of molecular interrogation using single cell RNAseq to better understand the underlying changes at the genomic level, leading to the failure of neural tube closure (Ishihara et al., 2017). This will be an essential tool to developing intervention strategies to not only prevent preventable birth defects, but also to create essential therapeutic approaches to minimizing the morbidities associated with spina bifida. Finally, the importance of environmental influences on the 3D organization of DNA cannot be overlooked. Topologically associating domains (TADs) are genomic regions that represent potentially compromised structures that can be altered by a range of teratogens and are amenable to future investigations that may change the way we have been thinking about environmentally-induced birth defects (Liu and Wang, 2019).

## Concluding Remarks

NTDs represent one of many excellent examples of complex congenital malformations that result for as yet undefined interactions between genes and environmental teratogens. Efforts to define such interactions and clearly identify the sensitivity or susceptibility genes have been slow to evolve. However, recent development of advanced DNA sequencing tools at reasonable experimental costs have created new possibilities that this vexing data gap might soon be resolved. Like many investigators, we look forward to the continued evolution of scientific technologies and approaches that help us to understand the genetic architecture of NTDs and biomechanical events responsible for these devastating birth defects. We look forward to the day when preventable birth defects can indeed be prevented.

## Author Contributions

RF and CC primarily written the manuscript. RF, BW, XC, YL, JS, S-EK, GT, YL, and RC participated in editing and revising the draft manuscript. RF, BW, RC, XC, and YL generated the data for the manuscript. RC, XC, S-EK, and GT generated the figures. All authors contributed to the article and approved the submitted version.

### Conflict of Interest

RF, RC, and BW once held leadership positions with the now dissolved TeratOmic Consulting LLC. RF also receives travel funds to attend the editorial board meetings of the *Journal of Reproductive and Developmental Medicine* published out of the Red Hospital of Fudan University.

The remaining authors declare that the research was conducted in the absence of any commercial or financial relationships that could be construed as a potential conflict of interest.
